# The Value of Haematological Parameters and Tumour Markers in the Prediction of Intestinal Obstruction in 1474 Chinese Colorectal Cancer Patients

**DOI:** 10.1155/2020/8860328

**Published:** 2020-08-14

**Authors:** Yinghao Cao, Songqing Ke, Junnan Gu, Fuwei Mao, Shuang Yao, Shenghe Deng, Lizhao Yan, Ke Wu, Li Liu, Kailin Cai

**Affiliations:** ^1^Department of Gastrointestinal Surgery, Union Hospital, Tongji Medical College, Huazhong University of Science and Technology, Wuhan, Hubei 430022, China; ^2^Department of Epidemiology and Biostatistics, The Ministry of Education Key Lab of Environment and Health, School of Public Health, Tongji Medical College, Huazhong University of Science and Technology, Wuhan, Hubei 430022, China

## Abstract

Intestinal obstruction, a life-threatening problem, often occurs in patients with advanced colorectal cancer (CRC). However, the cause of obstruction is still unknown. Very few prediction models for intestinal obstruction in CRC exist, and their results are unreliable. Therefore, we investigated whether preoperative serum tumour markers (STMs) combined with haematological and biochemical markers could be used as predictors. We retrospectively analysed 1474 patients with CRC who underwent radical resection after admission. Several clinical features, STMs, and serum biochemical and haematological indicators were analysed. Predictors of intestinal obstruction were analysed with univariate and multivariate logistic regression. The accuracy of the multivariate predictors of obstruction was measured by the area under the receiver operating characteristic (ROC) curve (AUC). The Kaplan-Meier method was used to create survival curves. Obstruction was found more in males (62.18%), never-smokers (73.95%), the left colon (54.20%), the tumour diameter > 4.5 cm (55.88%), high differentiation (89.50%), and negative nerve invasion (70.17%). The serum tumour markers (STMs) and peripheral blood routine indexes (PBRI) were significantly associated with obstructive status (*p* < 0.05). Multivariate analysis demonstrated that the neutrophil and lymphocyte counts, carcinoembryonic antigen, carbohydrate antigen 19-9, carbohydrate antigen 125, albumin, alkaline phosphatase, gamma-glutamyl transpeptidase, total protein, and neutrophil-to-lymphocyte ratio were predictors of intestinal obstruction (*p* < 0.05). The AUC for the curve with all the eight factors was 0.715 (95% confidence interval: 0.673-0.758). The STMs and PBRI were related to the obstruction status of the patients, and they could be used in combination with other clinical factors to significantly improve diagnosis and management of intestinal obstruction in CRC patients.

## 1. Introduction

Colorectal cancer (CRC) is one of the most common malignant diseases. In 2018, it was the third most common cancer and second leading cause of mortality worldwide as over 1.8 million new cases and 881 000 deaths were recorded [1]. Partial or complete intestinal obstruction occurs in 7% to 29% of all CRC patients, with about 70% of these cases occurring in the left colon. Fatal complications are likely to occur if left untreated [2–5]. Intestinal obstruction is a clinical emergency. Due to the poor conditions and inadequate intestinal preparation in patients with obstruction, the risk of surgery, postoperative complications, and mortality were extremely high compared with the elective surgery [6, 7]. The world guidelines for emergency surgery point out that intestinal obstruction caused by CRC can be dredged first, after which radical tumour resection can be performed at another time while direct radical resection can be performed for patients with nonobstructive CRC [8]. Early identification of obstruction is critical to the patient's treatment.

Relevant research also showed that timely and effective surgery has a better effect on intestinal obstruction caused by CRC [9–11]. The cause of obstruction caused by tumour growth is still unknown, and there is no relevant prediction model for the determination of preoperative patient obstruction. The purpose of this study was to identify predictors of intestinal obstruction due to CRC growth.

## 2. Materials and Methods

### 2.1. Selected Patients and Study Design

This study was approved by the Ethics Committee of the Tongji Medical College, Huazhong University of Science and Technology. Between January 2015 and December 2017, 1604 CRC patients complicated or not with intestinal obstruction consulted at the Wuhan Union Medical College Hospital, and they were all diagnosed on admission. We retrospectively collected the haematological, biochemical, and serum tumour marker (STM) information of the patients. All patients were tested for at least one STM including carcinoembryonic antigen (CEA), cancer antigen (CA) 125, and CA 19-9. Haematological and biochemical parameters including the total neutrophil (NEU) count, white blood cell (WBC) count, serum albumin (ALB), alkaline phosphatase (ALP), gamma-glutamyl transpeptidase (GGT), and serum total protein level (Tp) were measured before surgery. The preoperative neutrophil-to-lymphocyte ratio (NLR) was calculated as the neutrophil count divided by the lymphocyte (LYM) count. We built an Internet big data platform and started data collection for all patients in 2017 (national software copyright 2019SR1048616). We made a definitive diagnosis of all CRC patients in the database according to international standards.

The medical records of all patients were reviewed. Their tumour stages were classified based on the seventh edition of the American Joint Committee on Cancer (AJCC) staging system and Union for International Cancer Control (UICC) [12]. A total of 130 patients with the following conditions were excluded from the study: (1) history of abdominal surgery (*n* = 98); (2) history of another cancer (*n* = 24); (3) multiple tumours with inconsistent pathology results (*n* = 12); and (4) intestinal perforation (*n* = 5).

### 2.2. Haematological, Biochemical, and STM Measurement

Haematological and biochemical parameters were done using an automated blood analyser. When the results were doubtful, manual counting correction was done by the laboratory physician. A chemiluminescent immunoassay kit was used for the measurement of STMs (Abbott Laboratories, I4000, America). Blood samples were obtained from all patients by peripheral venous puncture before any anticancer therapy. The sensitivity of the NEU count, WBC count, LYM count, ALB, ALP, GGT, Tp, CEA, CA 19-9, and CA 125 was 3.5%-7.5%, 0.18%, 0-13.8%, 1.43%, 1.36 ng/mL, 3.3 U/L, 0.76 g/dL, 0.5 ng/mL, 2 ng/mL, and 1 ng/mL, respectively. Their imprecision was NEU ≤ 3%; WBC ≤ 3%; LYM ≤ 3%; ALB ≤ 2%; ALP ≤ 6.2%; GGT ≤ 4.8%; Tp ≤ 3%; CEA ≤ 5%; CA 19‐9 ≤ 3.3%, and CA 125 ≤ 6%. Their respective cut-off values were as follows: NEU, 1.8-6.3 G/L; WBC, 3.5-9.5 G/L; LYM, 1.1-3.2 G/L; ALB, 35-55 g/L; ALP, 40-150 U/L; GGT, 11-50 U/L; Tp, 64-83 g/L; CEA < 5.0 *μ*g/L; CA 19‐9 < 37 U/mL, and CA 125 < 35 U/mL. Three types of collection tubes were used in this study. The first one was the purple vacuum blood collection tube with anticoagulant (ethylenediaminetetraacetic acid- (EDTA-) K2 or K3) in which whole blood was collected, and the haematology parameters (NEU, WBC, LYM counts, etc.) were studied. The second one was the red vacuum blood collection tube without anticoagulant in which serum was collected, and the biochemical parameters (ALB, ALP, GGT, and Tp) were studied. The third one was the yellow Clot Activator and gel-glass vacuum blood collection tube in which serum was collected, and the tumour markers (CEA, CA 19-9, and CA 125) were tested.

### 2.3. Follow-Up after Surgery and Postoperative Chemotherapy

A total of 236 patients in the obstructive colorectal cancer (OCC) group and 1236 patients in the nonobstructive colorectal cancer (non-OCC) group were followed up for 5 years. All serum and blood tests were done every 3 months, computed tomography (CT) every 6 months, and colonoscopy every 12 months. When we suspected recurrence, CT and positron emission tomography were performed at that time. Patients with stage II or III disease were administered oral S-1 or capecitabine for 6 months after surgery while those with stage IV disease were placed on intensive chemotherapy for six months after surgery, depending on their physical status.

### 2.4. Statistical Analysis

The categorical variables were expressed as number (percentage) while the continuous variables were expressed as mean ± standard deviation. Receiver operating characteristic (ROC) curves were applied to transform the continuous variables (NEU, WBC, LYM, CEA, CA 19-9, CA 125, ALB, ALP, GGT, Tp, and NLR) into dichotomized variables by using inflexion points as cut-offs (Table 1). Univariate and multivariate logistic regression was used to analyse the relationship between the clinical features, haematological and biochemical parameters, and STM levels and obstruction. The accuracy of the multivariate predictors of obstruction was measured by the area under the ROC curve (AUC). The Kaplan-Meier method was used to create survival curves of disease-free survival (DFS) and overall survival (OS) after surgery, and different groups were compared using the log-rank analysis. Subgroup analyses were conducted to show the prognostic association with individual clinical indicators among patients with different features, and the results were presented as forest plots. All statistical analyses were performed using SAS 9.4 (SAS Institute Inc., Cary, North Carolina, USA) and R3.5.1 (R Foundation for Statistical Computing, Vienna, Austria). Two-sided *p* < 0.05 was statistically significant.

## 3. Results

### 3.1. Patient Clinical Characteristics

**Figure 1:**
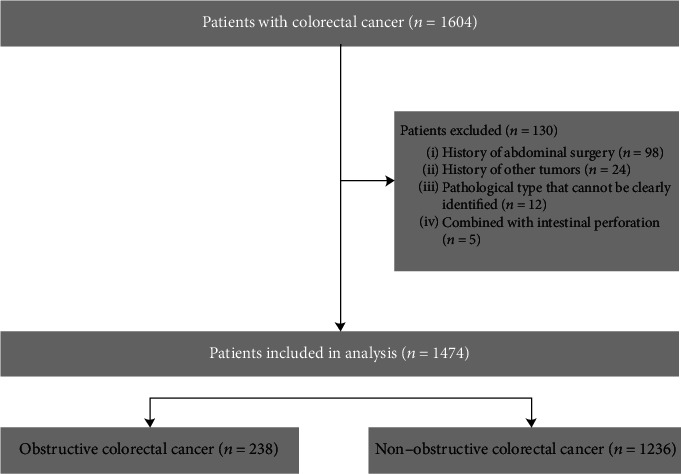
Strategies for selecting patients to be included in the study.

A total of 1474 CRC patients were included in our study between January 2015 and December 2017 (Figure 1). The OCC group was made up of 238 patients (16.01%), and their median age was 59 (21-86) years. The non-OCC group was made up of 1236 patients (83.99%), and their median age was 58.5 (22-92) years. There was no significant difference between the two groups (*p* > 0.05) (Table 2).

### 3.2. Comparison of Clinical Features between the OCC and Non-OCC Groups

The following were the features in both groups (OCC vs. non-OCC patients): male (62.18% vs. 37.82%), never-smokers (26.05% vs. 73.95%), left colon (54.20% vs. 45.80%), tumour diameter exceeds 4.5 cm (55.88% vs. 44.12%), low differentiation (89.50% vs. 10.50%; *p* = 0.0044), patients with negative nerve invasion (70.17% vs. 29.83%), low lymphocyte (1.30 ± 0.75 vs. 1.55 ± 0.68; *p* < 0.001), high CEA (5.23 ± 12.40 vs. 3.30 ± 6.30; *p* < 0.001), high CA 19-9 (11.60 ± 37.10 vs. 8.65 ± 18.50; *p* < 0.001), high CA 125 (20.75 ± 35.10 vs. 11.40 ± 8.30; *p* < 0.001), high NLR (2.78 ± 2.71 vs. 2.28 ± 1.58; *p* < 0.001), low ALB (38.15 ± 6.50 vs. 40.90 ± 6.10; *p* < 0.001), low ALP (71.50 ± 27.00 vs. 75.00 ± 27.50; *p* = 0.0403), and low Tp (61.95 ± 9.80 vs. 65.30 ± 9.40; *p* < 0.001). 44.54% of patients in the OCC group received chemotherapy and 5.04% received radiotherapy. In the non-OCC group, 54.69% received chemotherapy and 5.74% radiotherapy. All patients received first-line chemotherapy, and none received bevacizumab or cetuximab. There were no significant differences in age, gender, tumour history, circumferential margin, NEU count, WBC count, and GGT (Table 2).

### 3.3. The Obstructive Status Can Be Predicted by STMs Combined with Clinical Features

**Figure 2:**
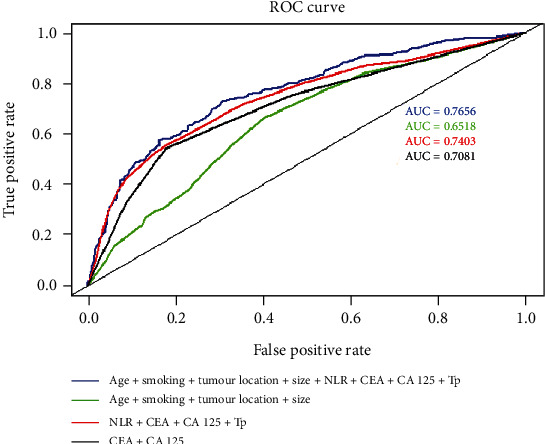
ROC curves of the combination of features for predicting obstruction status. The green line includes age, smoking status, tumour location, and tumour size; the dark line includes CEA and CA 125; the red line includes NLR, CEA, CA 125, and Tp, with AUCs of 0.6518, 0.7081, and 0.7403, respectively. When these factors were used together (age, smoking, tumour location, tumour size, NLR, CEA, CA 125, and Tp), the AUC was 0.7656.

The univariate logistic regression analysis showed that NEU, WBC, and LYM counts; CEA, CA 19-9, CA 125, ALB, ALP, GGT, and Tp levels; and NLR were significantly correlated with OCC. High NEU count (OR, 1.866; *p* < 0.001), high WBC count (OR, 1.574; *p* = 0.006), high LYM count (OR, 0.437; *p* < 0.001), high CEA (OR, 1.923; *p* < 0.001), high CA 19-9 (OR, 2.077; *p* < 0.001), high CA 125 (OR, 5.466; *p* < 0.001), low ALB (OR, 0.340; *p* < 0.001), low ALP (OR, 0.671; *p* = 0.009), low GGT (OR, 1.830; *p* = 0.0247), low Tp (OR, 0.402; *p* < 0.001), and NLR (OR, 2.268; *p* < 0.001) were important predictors of OCC. The cut-off value of NLR was 3.064, (Table 1). When these factors were used together (age, smoking, tumour location, tumour size, NLR, CEA, CA 125, and Tp), the AUC was 0.7656 (Figure 2). The multinomial logistic regression analysis showed that obstructive status was associated with different haematological and biochemical parameters and STMs (Table 1).

### 3.4. The Subgroup Analysis

**Figure 3:**
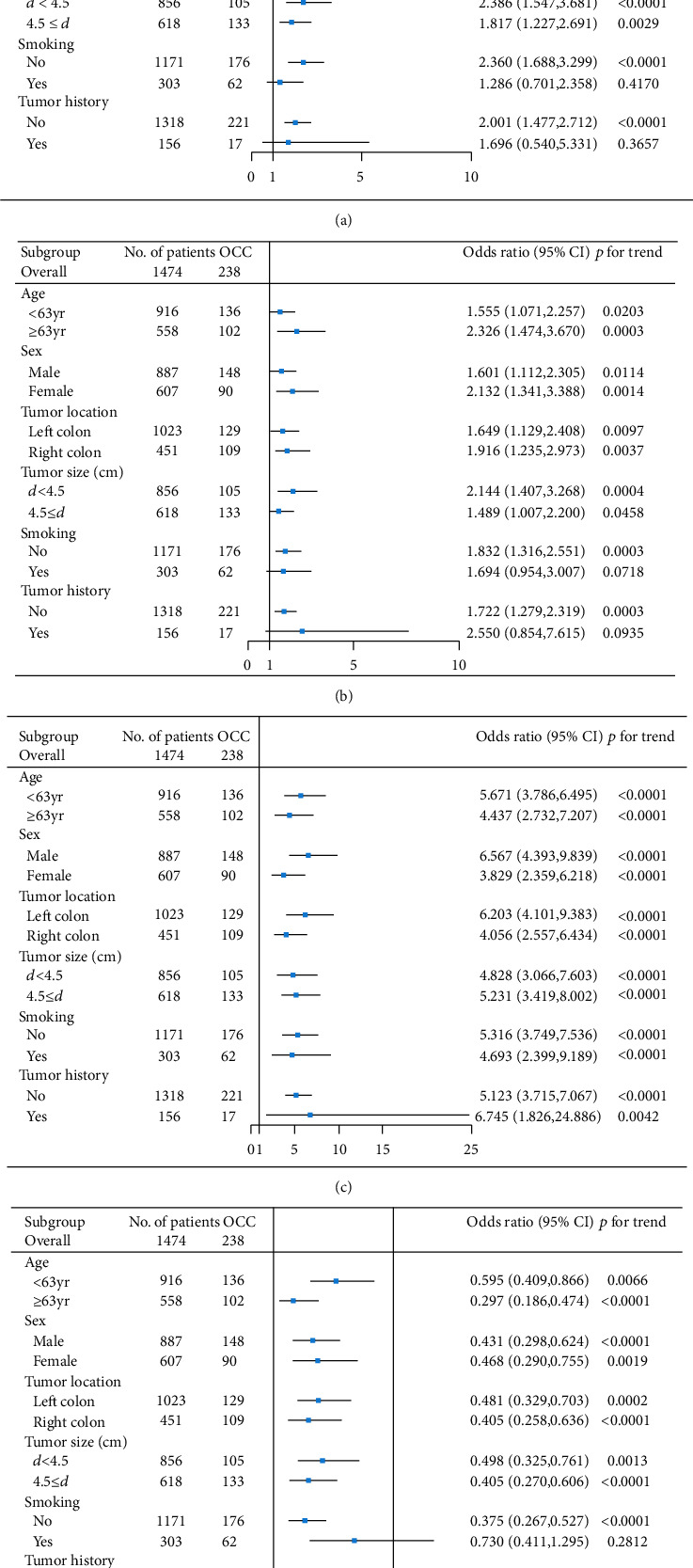
The forest plot of the impact of different factors on the colorectal cancer patients in different subgroups. (a) A forest plot of the impact of NLR on the colorectal cancer patients in different subgroups. (b) A forest plot of the impact of CEA on the colorectal cancer patients in different subgroups. (c) A forest plot of the impact of CA 125 on the colorectal cancer patients in different subgroups. (d) A forest plot of the impact of Tp on the colorectal cancer patients in different subgroups.

We divided the CRC patients into different subgroups according to their age, gender, tumour location, tumour size, smoking status, and family history of cancer. CEA was a risk factor within all the subgroups except in the family history of cancer subgroups (Figure 3(a)). CA 125 was a strong risk factor within all subgroups (*p* < 0.0001, Figure 3(b)). Tp was a protective factor within all the subgroups except in the family history of cancer subgroups (Figure 3(c)). NLR was a risk factor within all the subgroups except in the family history of cancer subgroups (Figure 3(d)).

### 3.5. Comparison of DFS between the OCC and Non-OCC Groups with All Patients, Stage II and III CRC

**Figure 4:**
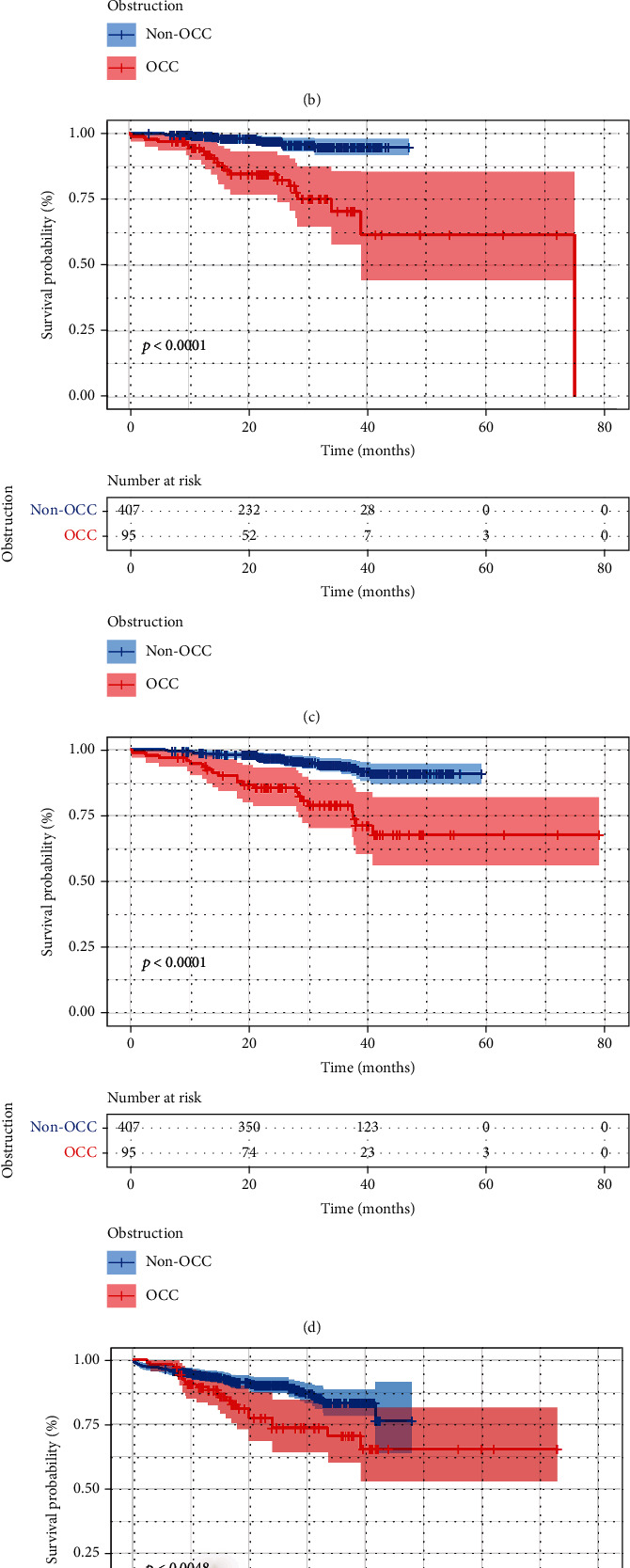
Comparison of DFS and OS between the OCC and non-OCC groups. (a) Comparison of DFS between the OCC and non-OCC groups. (b) Comparison of OS between the OCC and non-OCC groups. (c) Comparison of DFS between the OCC and non-OCC groups in stage II. (d) Comparison of OS between the OCC and non-OCC groups in stage II. (e) Comparison of DFS between the OCC and non-OCC groups in stage III. (f) Comparison of OS between the OCC and non-OCC groups in stage III.

The 3-year DFS of the OCC group (*n* = 238) was 65.47% and that of the non-OCC group (*n* = 1236) was 81.68% (*p* < 0.001, Figure 4(a)).

For the patients with stage II CRC, the 3-year DFS of the OCC group (*n* = 95) was 70.35% and that of the non-OCC group (*n* = 407) was 94.85% (*p* < 0.001, Figure 4(c)).

For the patients with stage III CRC, the 3-year DFS of the OCC group (*n* = 106) was 70.61% and that of the non-OCC group (474) was 83.32% (*p* < 0.001, Figure 4(e)).

### 3.6. Comparison of OS between the OCC and Non-OCC Groups in All the Patients, Stage II and III CRC

The 3-year OS of the OCC patients (*n* = 238) was 68.52% and that of the non-OCC patients (*n* = 1236) was 82.25% (*p* < 0.001, Figure 4(b)).

For patients with stage II CRC, the 3-year OS of the OCC group (*n* = 95) was 78.88% and that of the non-OCC group (*n* = 407) was 93.58% (*p* < 0.001, Figure 4(d)).

For patients with stage III CRC, the 3-year OS of the OCC patients (*n* = 106) was 65.55% and that of the non-OCC patients (*n* = 474) was 83.17% (*p* < 0.001, Figure 4(f)).

## 4. Discussion

Many researchers have accepted that inflammatory response plays a dual role in tumour development. Firstly, chronic inflammatory responses trigger local accumulation of monocytes, platelets, and neutrophils that secrete cytokines to induce tumour angiogenesis and metastasis. Secondly, the increase in monocytes and lymphocytes creates a resistance to tumour invasion [13]. Vakkila et al. suggested that inflammation is related not only to carcinogenesis but also to cancer progression [14, 15]. Tumour growth is facilitated by the release of inflammatory cytokines and chemokines by the invading WBCs, which are themselves stimulated by the tumour. Therefore, C-reactive protein, interleukin-6, and other inflammatory markers are elevated in various malignant diseases, which are closely related to the prognosis of patients with malignant diseases [16, 17]. There is increasing evidence that elevated levels of NLR (biomarker of inflammation) are associated with poor prognosis for ovarian cancer, cholangiocarcinoma, and CRC [18–21].

The combination of imaging and endoscopic diagnoses is the main method for preoperative diagnosis of intestinal obstruction. However, China has a large population, medical resources are unevenly distributed in various regions, and the level of endoscopy varies from hospital to hospital at relatively high inspection costs. This results in some patients lacking imaging and endoscopic diagnoses. Patients do not come to the hospital until the obstruction is complete. This not only makes the follow-up treatment more troublesome but also greatly reduces the postoperative biochemical treatment for intestinal obstruction patients since most of them require an ostomy. Although there is a barrier in imaging and endoscopy, country or provincial hospitals can quickly and accurately test haematological and biochemical parameters and STMs at low costs. A relationship has been established between these and obstruction [22]. Therefore, it is feasible to use them as obstruction predictors.

In this study, the inflammatory marker levels (NEU count, LYM count, and NLR) in the OCC group were significantly higher than those in the non-OCC group; however, there was no significant difference in the WBC count between the two groups. This is a particular malignant disease phenomenon, unlike in inflammatory diseases caused by infection. OCC is often accompanied by severe local and systemic inflammatory reactions. Several causes have been identified including overgrowth of gut bacteria through displacement of swollen colon walls and septic shock. In this study, we found that the cut-off point for NLR was 3.064, which supports the presence of severe systemic inflammation. NLR was associated with intestinal obstruction due to CRC growth.

Malnutrition characterized by hypoproteinaemia is associated with poor long-term outcomes, and these markers have been shown to predict long-term outcomes of various malignancies [23, 24]. This was calculated by ALB, ALP, GGT, and Tp and the peripheral NEU, LYM, and platelet counts. In our study, the Tp and Alb levels of the OCC group were significantly lower than those of the non-OCC group.

Eto et al. suggested that there was no statistically significant difference in serum CEA and CA 19-9 levels between the OCC and non-OCC patients [22]. On the contrary, our results showed that serum CEA, CA 19-9, and CA 125 levels of the OCC group were significantly higher than those of the non-OCC group. This difference could be due to their small sample size.

We also demonstrated that the obstructive status can be predicted by combining clinical characteristics, haematological and biochemical parameters, and STM levels. Multivariate analysis demonstrated that NEU count, LYM count, CEA, CA 19-9, CA 125, ALB, ALP, and Tp were predictors of obstructive status. When NLR, CEA, CA 125, and Tp were used together, the AUC of the ROC curve was 0.7403, indicating that the ability to predict the obstructive status was superior to the previously identified correlation factors when combined with the level of relevant STMs such as CEA and CA 125 (AUC = 0.7081).

Previous studies have reported that patients with OCC have a significantly worse oncological outcome than patients with non-OCC [25]. In this study, a significant difference in the 3-year DFS and OS was observed between the OCC and non-OCC groups. At the same time, the OS and DFS in the OCC group were significantly reduced in stages II and III compared with those in the non-OCC group, and this outcome was consistent with those reported previously [26, 27]. We also found that our results were different from the study by Eto et al., and it could be mainly due to their small sample size. A large-scale case control is needed to explore whether STMs and clinical characteristics can be used to predict the obstructive status, thereby assisting clinicians in managing these patients.

At the same time, this study had several limitations. First, it was a retrospective design with its inherent limitations. Second, we did not assess the impact of other therapies on survival in the two groups.

In conclusion, intestinal obstruction can be predicted by haematological and biochemical parameters, STM levels, and clinical factors in CRC patients. Our results suggested that preoperative NLR could be a useful predictor of bowel obstruction due to CRC growth.

## Figures and Tables

**Table 1:** The univariate and multinomial logistic regression analysis between high and low levels groups of biomarkers.

	Totle patients				Univariate	*P*	Multivariate	*P*
		Male	Cut-point	AUC	OR (95% CI)		OR (95% CI)	
NEU	1474	867	4.82	0.5396	1.866 (1.378,2.528)	<0.0001	1.593 (1.163,2.181)	0.0037
WBC	1474	867	7.57	0.5129	1.574 (1.139,2.175)	0.0060	1.371 (0.982,1.915)	0.0640
LYM	1474	867	1.38	0.6174	0.437 (0.330,0.579)	<0.0001	0.444 (0.332,0.592)	<0.0001
CEA	1474	867	4.84	0.6004	1.923 (1.454,2.544)	<0.0001	1.778 (1.336,2.366)	<0.0001
CA19-9	1474	867	29.9	0.5475	2.077 (1.530,2.819)	<0.0001	1.975 (1.442,2.704)	<0.0001
CA125	1474	867	19.5	0.7005	5.466 (4.075,7.331)	<0.0001	5.148 (3.781,7.009)	<0.0001
ALB	1474	867	40.8	0.6573	0.340 (0.249,0.464)	<0.0001	0.392 (0.285,0.539)	<0.0001
ALP	1474	867	63	0.5419	0.671 (0.497,0.904)	0.0088	0.619 (0.455,0.842)	0.0023
GGT	1474	867	67	0.5037	1.830 (1.080,3.101)	0.0247	1.712 (0.995,2.944)	0.0520
Tp	1474	867	62.8	0.6403	0.402 (0.303,0.532)	<0.0001	0.448 (0.335,0.598)	<0.0001
NLR	1474	867	3.064	0.6073	2.268 (1.709,3.010)	<0.0001	2.049 (1.530,2.743)	<0.0001

OR, odds ratio; 95% CI, 95%Confidence interval. Multivariate analysis adjusted for age, tumor location, tumor size and smoke. NEU, neutrophil; WBC, white blood cell; LYM, lymphocyte; CEA, carcinoembryonic antigen; CA19-9, carbohydrate antigen 19-9; CA125, cancer antigen 125; ALB, albumin; ALP, alkaline phosphatase; GGT, gamma-glutamyl transpeptidase; Tp, total protein; NLR = neutrophil/lymphocyte.

**Table 2:** Baseline Clinicopathologic Characteristics of colorectal cancer patients.

		OCC	Non-OCC	*P* ^∗^
		(n =238) (%)	(n =1236) (%)	
Age(yr) (median ± qrange)		59.00 ± 15.00	58.50 ± 16.00	0.1162
Sex	Male	148 (62.18)	719 (58.17)	0.2493
	Female	90 (37.82)	517 (41.83)	
Smoke	No	176 (73.95)	995 (80.50)	0.0220
	Yes	62 (26.05)	241 (19.50)	
Tumor history	No	221 (92.86)	1097 (88.75)	0.0595
	Yes	17 (7.14)	139 (11.25)	
Tumor location	Left colon	129 (54.20)	894 (72.33)	<0.0001
	Right colon	109 (45.80)	342 (27.67)	
TNM	I	4 (1.68)	193 (15.61)	<0.0001
	II	95 (39.92)	407 (32.93)	
	III	106 (44.54)	474 (38.35)	
	IV	33 (13.87)	162 (13.11)	
Tumor size (cm)	d <4.5	105 (44.12)	751 (60.76)	<0.0001
	4.5 ≤ d	133 (55.88)	485 (39.24)	
Differentiation	Low	25 (10.50)	69 (5.58)	0.0044
	High	213 (89.50)	1167 (94.42)	
Circumferential margin	No	236 (99.17)	1224 (99.03)	0.8492
	Yes	2 (0.83)	12 (0.97)	
Vascular tumor thrombus	No	187 (78.57)	1022 (82.69)	0.1301
	Yes	51 (21.43)	214 (17.31)	
Nerve invasion	No	161 (70.17)	988 (79.94)	0.0008
	Yes	71 (29.83)	248 (20.06)	
Chemotherapy	No	132 (55.46)	560 (45.31)	0.0040
	Yes	106 (44.54)	676 (54.69)	
Radiotherapy	No	226 (94.96)	1165 (94.26)	0.6669
	Yes	12 (5.04)	71 (5.74)	
NEN(G/L) (median ± qrange)		3.79 ± 2.53	3.59 ± 1.77	0.0526
WBC(G/L) (median ± qrange)		5.80 ± 2.97	5.84 ± 2.23	0.5276
LYM(G/L) (median ± qrange)		1.30 ± 0.75	1.55 ± 0.68	<0.0001
CEA(ug/L) (median ± qrange)		5.23 ± 12.40	3.30 ± 6.30	<0.0001
CA19-9(U/ml) (median ± qrange)		11.60 ± 37.10	8.65 ± 18.50	0.0201
CA125(U/ml) (median ± qrange)		20.75 ± 35.10	11.40 ± 8.30	<0.0001
ALB(U/L) (median ± qrange)		38.15 ± 6.50	40.90 ± 6.10	<0.0001
ALP(U/L) (median ± qrange)		71.50 ± 27.00	75.00 ± 27.50	0.0403
GGT(U/L) (median ± qrange)		18.00 ± 15.00	19.00 ± 13.00	0.8573
Tp(U/L) (median ± qrange)		61.95 9.80	65.30 ± 9.40	<0.0001
NLR (median ± qrange)		2.78 ± 2.71	2.28 ± 1.58	<0.0001

^∗^ *P* was calculated by the Wilcoxon test for continuous variables and the chi-square test for categorical variables. NEU, neutrophil; WBC, white blood cell; LYM, lymphocyte; CEA, carcinoembryonic antigen; CA19-9, carbohydrate antigen 19-9; CA125, cancer antigen 125; ALB, albumin; ALP, alkaline phosphatase; GGT, gamma-glutamyl transpeptidase; Tp, Total protein; NLR = neutrophil/lymphocyte.

## Data Availability

The data used to support the findings of this study are available from the corresponding author upon request.
